# Exploring the landscape of bariatric surgery in Africa: current provisions, challenges, and future prospects

**DOI:** 10.1097/MS9.0000000000002381

**Published:** 2024-07-17

**Authors:** Hareesha R. Bharadwaj, Muhammad Hamza Shah, Matan Bone, Priyal Dalal, Khabab Abbasher Hussein Mohamed Ahmed

**Affiliations:** a Faculty of Biology, Medicine and Health, The University of Manchester, Manchester; b School of Medicine, Queen’s University Belfast, Belfast; c Faculty of Biology, Medicine and Health, The University of Manchester, Manchester; d University of Central Lancashire, Preston, United Kingdom; e Faculty of Medicine, University of Khartoum, Khartoum, Sudan

Highlights

- This study examines the state of bariatric surgery in Africa, emphasising the obstacles, opportunities, and recent advancements in the field.
- Although bariatric surgery is widely accepted, little is known about how it is integrated into low-income countries and middle-income countries, especially in Africa.
- With an analysis of the available resources, an examination of the difficulties, and recommendations for solutions, the editorial seeks to close this gap.

Bariatric surgery is widely acknowledged as a pivotal intervention for severe obesity, consistently demonstrating significant efficacy in achieving sustained weight loss and alleviating associated comorbidities. For individuals with morbid obesity, these surgical procedures stand out as the most effective approach to achieving substantial and enduring weight reduction, while also addressing health complications and reducing mortality risks^1^. Despite the widespread integration of bariatric surgery in high-income nations, its adoption in low and middle-income countries, particularly in Africa, remains relatively underexplored. The increasing prevalence of chronic diseases like ischaemic heart disease and diabetes mellitus in Africa underscores the urgent need for effective obesity-related interventions^2^. However, there exists a notable gap in the literature regarding the adoption and implementation of bariatric surgical practices in these regions, highlighting the necessity for comprehensive research.

## Current standing

African nations, particularly in North Africa such as Egypt, demonstrate a dynamic interest in bariatric surgery research and innovation. There is a notable trend towards adopting gastric restrictive procedures like vertical banded gastroplasty and Roux-en-Y gastric bypass through mini-incisions, addressing concerns such as cost and time associated with laparoscopic surgeries while maintaining comparable benefits in operative efficiency and recovery times^3^. Comparative studies by Egyptian researchers indicate similar outcomes across various procedures such as laparoscopic sleeve gastrectomy, laparoscopic gastric greater curvature plication, and laparoscopic sleeve gastrectomy with loop bipartition, underscoring the robustness of these surgical options^4,5^. Furthermore, ongoing efforts in Egypt focus on refining techniques like laparoscopic sleeve gastrectomy with integrated gastropexy, which has shown promise in reducing postoperative symptoms and hospital readmissions^6^. These innovations contribute to improving the overall effectiveness and safety of bariatric surgeries in the region.

There has also been a continued focus on bariatric surgical procedural innovation, as exemplified by Attia^7^ investigation into the efficacy of laparoscopic sleeve gastrectomy with simultaneous crural repair in treating morbid obesity associated with gastroesophageal reflux disease. In South Africa, where obesity rates are the highest in Sub-Saharan Africa, proactive steps are being taken to integrate bariatric surgical services into mainstream obesity management practices. Lubbe *et al*.^8^ study demonstrates that the short-term safety and efficacy outcomes of Roux-en-Y gastric bypass and sleeve gastrectomies performed in the country were on par with international reports. This reflects African nations’ commitment to innovate, evaluate, and integrate bariatric surgical practices into broader healthcare services, addressing regional challenges posed by obesity.

Economic evaluations are emerging as a critical aspect of integrating bariatric surgery into mainstream healthcare provision. In Tunisia, studies assess the cost-effectiveness of gastric bypass and sleeve gastrectomy, utilising a Markov model to demonstrate economic viability compared to conventional obesity treatments. The analysis presents favourable incremental cost-effectiveness ratios and substantial gains in quality-adjusted life years, emphasising its importance in the region^9^. Similarly, in Cameroon, research explores the incorporation of sleeve gastrectomy into resource-limited settings, indicating its efficacy and economic feasibility^10^. The research landscape actively investigates the long-term effects of bariatric surgery and addresses associated complications. Efforts also focus on nutritional assessments for populations undergoing metabolic surgery, recognising challenges in resource-constrained environments^11^. Comprehensive evaluations have been conducted on bariatric surgery’s long-term effects on vitamin D, parathormone, and serum calcium levels^12^. These research endeavours continue to shape the trajectory of bariatric surgery in Africa, paving the way for effective and context-specific interventions in the dynamic healthcare landscape.

### Challenges

Advancement of bariatric and metabolic surgery in Africa faces significant challenges across multiple fronts. Primarily, the inadequacy of healthcare infrastructure in many African countries severely limits the widespread implementation of bariatric procedures, as these nations often lack the necessary facilities and equipment for specialised surgeries. Moreover, there is a critical shortage of healthcare professionals trained in bariatric and metabolic surgery, which extends beyond surgeons to encompass all medical staff involved in patient care, further hindering effective programme establishment and sustainability^13^. Economic factors also play a pivotal role in restricting the growth of bariatric surgeries in Africa. The high costs associated with procedures and postoperative care render them financially inaccessible for many patients, thereby straining healthcare resources, particularly in lower-income countries. Despite promising data from various single-centre studies on cost-effectiveness, there remains an urgent need for more extensive research to comprehensively elucidate this aspect^8^.

Public awareness and perceptions present additional challenges. There is insufficient knowledge among healthcare providers and the general population regarding the indications and outcomes of bariatric surgery. Moreover, there is a lack of comprehensive information on the positive impact of these surgeries on quality of life and body image, alongside a growing demand for body contouring procedures postsurgery. Furthermore, the mental health implications of bariatric surgery remain inadequately characterised, highlighting a significant gap in understanding the holistic effects of these procedures. Addressing these knowledge gaps through further research and awareness efforts is crucial^8,10,13^. Additionally, familial influences on eating and lifestyle habits significantly impact patient decisions to seek surgery, especially among women and children. Despite concerns, evidence does not suggest that fasting during Ramadan affects surgical outcomes. Challenges such as disordered eating behaviours, poor nutrition, and low dietary compliance also influence postsurgery weight loss outcomes. The role of physical activity and exercise in the postbariatric surgery context remains poorly understood^10,12,13^. Moreover, the regulatory and policy environment for bariatric and metabolic surgery in many African countries is underdeveloped, lacking clear and comprehensive guidelines. This deficiency poses risks to patient safety and compromises the quality of care provided. Addressing these multifaceted challenges is crucial for fostering the sustainable development of bariatric and metabolic surgery in the African context^7,8,10,13^.

### Future prospects

Looking ahead, overcoming the challenges in advancing bariatric and metabolic surgery in Africa requires a multifaceted approach. Collaborative efforts involving governments, international organisations, and the private sector could be pivotal in addressing the inadequacies of healthcare infrastructure. Investments in building and upgrading facilities, along with providing necessary equipment, would establish the foundation for widespread implementation of bariatric procedures. For this purpose, establishing targeted training programs for healthcare professionals in bariatric and metabolic surgery remains crucial. This initiative could involve partnerships with international institutions for knowledge exchange and the development of local training centres. It should extend beyond surgeons to include a broader spectrum of medical staff involved in patient care. Advocacy for increased research funding and financial support from governmental and nongovernmental entities could facilitate cost-effective strategies and interventions. A comprehensive and collaborative research approach is essential to address conflicting data on cost-effectiveness, laying a foundation for evidence-based decision^14^.

In addition, strategic public awareness campaigns tailored to cultural contexts can combat misconceptions and stigma surrounding obesity. Educational initiatives about obesity risks and surgical benefits should target diverse demographics, promoting better-informed public decisions and encouraging appropriate treatment-seeking behaviours. Recognising the influential role of familial influences, healthcare education initiatives can specifically target families in North Africa, fostering understanding and support within familial networks to improve healthcare decisions and practices and enhance patient outcomes^13^. Facilitating research collaboration and data sharing among African countries can address the lack of localised research on the outcomes of bariatric and metabolic surgeries. This collaborative approach can lead to a comprehensive understanding of unique challenges and effective interventions tailored to African populations. Advocacy for the development and implementation of clear and comprehensive guidelines for bariatric and metabolic surgery is crucial. Engaging policymakers and stakeholders in shaping regulatory environments will enhance patient safety and overall care quality.

## Ethical approval

Ethics approval was not required for this editorial article.

## Consent

Informed consent was not required for this editorial article.

## Source of funding

This study received no funding or financial support.

## Author contribution

H.R.B. and M.H.S.: conceived the idea, wrote the manuscript, edited it, critically revised it, contributed equally to this study, and are co-first authors; M.B., P.D., and K.A.H.M.A.: wrote the manuscript, edited and reviewed it. All authors read and approved the final version of the manuscript.

## Conflicts of interest disclosure

The authors report there are no competing interests to declare.

## Research registration unique identifying number (UIN)

1. Name of the registry: not required because this is an editorial.
2. Unique identifying number or registration ID: not applicable because this is an editorial.
3. Hyperlink to your specific registration (must be publicly accessible and will be checked): not applicable because this is an editorial.

## Guarantor

Khabab Abbasher Hussien Mohamed Ahmed, Faculty of Medicine, University of Khartoum, Khartoum 11111, Sudan. E-mail: khabab9722@gmail.com, ORCID: https://orcid.org/0000-0003-4608-5321

## Data availability statement

Data availability is not applicable to this article as no new data were created or analysed in this study.

## Provenance and peer review

Not commissioned, externally peer-reviewed.
